# Alcohol related dementia and Brain Imaging

**DOI:** 10.1192/j.eurpsy.2023.1095

**Published:** 2023-07-19

**Authors:** I. Faria, J. Galiano, B. Wildenberg, T. Silva, C. Silva

**Affiliations:** Psychiatry, Coimbra Hospital and University Centre, Coimbra, Portugal

## Abstract

**Introduction:**

Alcohol is considered a social evil worldwide owing to its vast array of associated problems and complications, which may manifest in medical, legal or social domains. Excessive and prolonged alcohol use may lead to permanent structural and functional damage to the brain. The evidence from neuroimaging, neuropathological reports and autopsy evaluations suggest some degree of brain pathology in individuals diagnosed with an alcohol related disorder.

**Objectives:**

The aim of this study is to characterize the structural imaging findings on computed tomography (CT) and magnetic resonance (MR) imaging of our sample and provide an overview of the literature on the subject.

**Methods:**

Retrospective observational study with inpatients of Coimbra Hospital and University Centre (CHUC), who had alcohol use disorder diagnosis associated with dementia or cognition deficit. Patients were admitted from 2017 to 2021 and submitted to neuroimaging: CT and MR. Data was collected in May 2021 at informatic system.

**Results:**

Among 38 participants, the median age was 64 years; 86,8% were male. 35 realize CT, 34 with alterations: 23 with microvascular lesions, 17 with cortical atrophy, 8 with white matter hypodensities and 7 with subcortical atrophy. From all patients, only 14 realize MR, 13 with alterations, the most common vascular leukoencephalopathy and cortical atrophy.

**Conclusions:**

Our results support the hypothesis of neuroimaging changes resulting from alcohol consumption. The severity of alcohol dependence also correlates with neuropathophysiological and neuroimaging changes. Volume shrinkage, altered glucose metabolism and perfusion along with evidence of markedly decreased neuron density are commonly reported. The evidence of neuro-circuit disturbances is seen in form of significant loss of white matter (most prominent in the prefrontal cortex, cerebellum and corpus callosum) on functional imaging. Greater cognitive impairment has been associated with multiple and repeated withdrawal due to greater neuronal damage, and can limit the psychotherapeutic intervention, the adherence to pharmacological therapy and abstinence maintenance. The sheer presence of alcohol use disorder should encourage a neuroimaging evaluation.

**Disclosure of Interest:**

None Declared

